# Preliminary Finnish Measures of Eating Competence Suggest Association with Health-Promoting Eating Patterns and Related Psychobehavioral Factors in 10–17 Year Old Adolescents

**DOI:** 10.3390/nu7053828

**Published:** 2015-05-21

**Authors:** Tilles-Tirkkonen Tanja, Nuutinen Outi, Suominen Sakari, Liukkonen Jarmo, Poutanen Kaisa, Karhunen Leila

**Affiliations:** 1 Institute of Public Health and Clinical Nutrition, University of Eastern Finland, PO Box 1627, 70211 Kuopio, Finland; E-Mails: outi.nuutinen@uef.fi (N.O.); kaisa.poutanen@vtt.fi (P.K.); leila.karhunen@uef.fi (K.L.); 2 Department of Public Health, University of Turku, 20014 Turku, Finland; E-Mail: suominen@utu.fi; 3 Department of Public Health, University of Skövde, PO Box 408, 54128, Skövde, Sweden; 4 Department of Sport Sciences, University of Jyväskylä, PO Box 35, 40014 Jyväskylä, Finland; E-Mail: jarmo.liukkonen@jyu.fi; 5 VTT—Technical Research Centre of Finland, PO Box 1000, 02044 VTT, Finland; 6 Institute of Clinical Medicine, Internal Medicine, Kuopio University Hospital, PO Box 100, 70029 KYS, Kuopio, Finland

**Keywords:** eating competence, eating patterns, adolescents, sense of coherence, self-esteem, body weight

## Abstract

Eating competence is an attitudinal and behavioral concept, based on The Satter Eating Competence Model. In adults, it has been shown to be associated with a higher quality of diet. Eating competence or its association with the quality of diet has not been studied in adolescents. The aim of the current study was to explore the utility of using a preliminary Finnish translation of the ecSI 2.0 for evaluating presumed eating competence and its association with food selection, meal patterns and related psychobehavioral factors in 10–17 year old adolescents. Altogether 976 10–17 years old Finnish adolescents filled in the study questionnaire. When exploring the construct validity of ecSI 2.0, the confirmatory factor analysis (CFA) indicated acceptable model fit and all four components of the ecSI 2.0 (eating attitudes, food acceptance, internal regulation of food intake, management of eating context) correlated with each other and were internally consistent. Over half (58%) of the adolescents scored 32 or higher and were thus classified as presumably eating competent (pEC). Eating competence was associated with greater meal frequency, more frequent consumption of vegetables and fruits, and more health-promoting family eating patterns. In addition the pEC, adolescents more often perceived their body size as appropriate, had less often tried to lose weight and had a higher self-esteem and a stronger sense of coherence than the not pEC ones. Family eating patterns and self-esteem were the main underlying factors of eating competence. In conclusion, this preliminary study suggests eating competence could be a useful concept to characterize eating patterns and related behaviors and attitudes in adolescents. However, these preliminary findings need to be confirmed in further studies with an instrument fully validated for this age group.

## 1. Introduction

Eating competence is an attitudinal and behavioral concept, which is based on The Satter Eating Competence Model (ecSatter). The ecSatter is a biopsychosocial model designed for use in nutrition education and for characterization of eating attitudes and behavior [1,2]. It is a practice-based model, informed and corrected by research observations [1]. It consists of four basic components. The first, eating attitudes, includes positive, relaxed and flexible interest in food and eating and responsive attunement to inner and outer experiences relative to eating. The second, food acceptance, denotes cognitive and behavioral processes and external influences of learning to accept and like a variety of foods, including new foods. The third, internal regulation of food intake, denotes to the experiential processes of hunger, appetite and satiety. It also includes acceptance of body weight that evolves from such internally regulated eating. The fourth, management of eating context, prioritizes structure and meal planning as well as permission to eat adequate amounts of preferred food at predictable times. The main tenets of ecSatter thus emphasize eating enjoyment, internal regulation of eating, body weight satisfaction regardless of real body weight, regular meal frequency and eating a variety of foods for pleasure rather than the necessity to meet dietary guidelines.

Two inventories have been developed to measure eating competence, the original ecSatter Inventory (ecSI) [3] and a further developed version ecSatter Inventory for Low-Income (ecSI/LI) [4], recently renamed as the Satter Eating Competence Inventory 2.0 (ecSI 2.0) [5]. Both ecSI and ecSI 2.0 have been validated in adult populations [3,4] and are shown to have a high congruence with each other [5,6]. The ecSI 2.0 has, however, been found to be more understandable [5,7] which supports the use of the ecSI 2.0 for a general audience [5]. Both of these inventories can be used to classify persons into two groups: eating competent (EC) and not eating competent (not EC). They have not previously been tested in adolescents.

Although ecSatter does not emphasize portion size and specific food or nutrient intake, eating competence has been associated with a higher quality of diet among adults [3,4,8,9,10]. Based on earlier studies, competent eaters consume fruits [3,8], vegetables [8], whole grains [8] and fish [8] more often and have greater adherence to a Mediterranean type of diet [9] than non-EC ones. They also have lower body mass index (BMI) and greater weight satisfaction [3]. EC parents consume breakfast and dinner more frequently with their children and provide better availability of vegetables and fruits at home [11]. Eating competence and its association with eating patterns, such as meal and snack routines and food choice, have not been previously studied in adolescents. It could be hypothesized that eating competence is also associated with health-promoting eating and related behavioral patterns in this age group. Poor quality of diet, overweight, body dissatisfaction and eating disorders have increased among adolescents in recent decades [12,13,14,15,16,17]. In addition, earlier prospective studies have shown that eating habits in childhood and adolescence appear stable and predict eating habits in later life [18]. Hence, factors related to the quality of diet and eating patterns in general need to be studied in adolescence since they are potential modifiers of eating-related health behavior in adulthood as well. New findings, by increasing the understanding of the topic, could then be applied in health promotion targeted for children and adolescents.

Family eating patterns and parenting practices, which influence development of overall eating patterns [17,19,20], could also affect eating competence. In addition, a reported association between eating competence and high self-esteem in adults [4] could be true for adolescents. Self-esteem, in turn, is associated with a sense of coherence (SOC) [21], a key theoretical construct in the salutogenic model [22,23] representing orientation to life. SOC is composed of three dimensions: comprehensibility (capacity to perceive the world and life events as understandable, ordered and predictable), manageability (confidence in one’s resources to deal successfully with environmental demands), and meaningfulness (belief that life is worthwhile and that challenges in life merit investment of effort and resources). SOC has been identified as an important factor influencing health behavior [24] and eating patterns in adults [25,26,27] and adolescents [28]. Also in our earlier study SOC was associated with adolescents’ eating patterns [29], and therefore we were interested in examining its role in this context as well.

Thus, the aim of the present study was to explore the utility of using a preliminary Finnish translation of the ecSI 2.0 for evaluating presumed eating competence and its association with food selection, meal patterns and related psychobehavioral factors in 10–17 year old adolescents.

## 2. Methods

### 2.1. Data Collection

**Figure 1:**
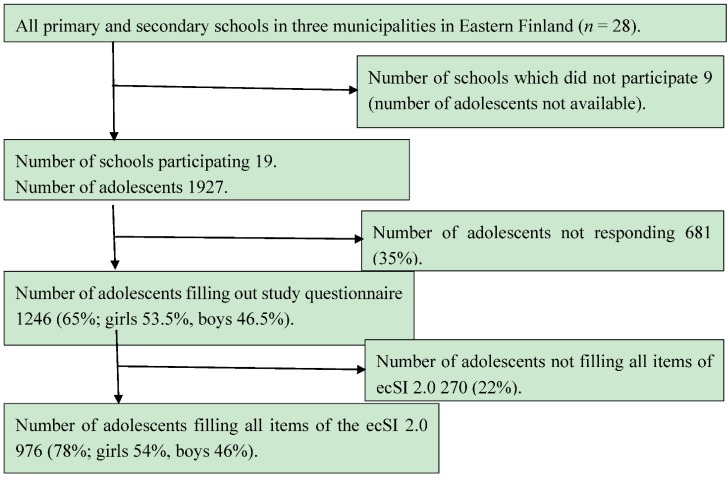
The study population.

In spring 2012, an invitation to participate in the study was sent to the principals of all primary and secondary schools in the towns of Kajaani and Kuhmo and the municipality of Sotkamo, located in Eastern Finland (Figure 1). In Sotkamo and Kuhmo, 12 schools out of 14, and in Kajaani seven schools out of 14, were willing to participate in the study. In the participating schools, students at grades five to nine (age range 10–17 years, *i.e.*, preadolescents and adolescents) were asked to complete individually a web-based questionnaire in a class during the school day. On average, it took 30 min to fill in the questionnaire. Teachers assisted the students with technical issues and with the interpretation of the questions, if needed. The questionnaire was open for approximately two weeks, during which the teachers took their classes to fill it in on a given day. Thus, finally altogether 1246 of the 1927 eligible adolescents filled in the questionnaire [667 (53.5%) girls and 579 (46.5%) boys]. Of these 1246, 78% *i.e.*, 976 responded to all items of the ecSI 2.0 [526 (54%) girls, 436 (46%) boys and 14 did not report their gender] and formed the study population. Adolescents who responded to all items of the ecSI 2.0 (*n* = 976) did not differ in gender, weight status, perception of body size, self-esteem or SOC from adolescents who did not answer all items of the ecSI 2.0 (*n* = 292). They were, however, older than those who did not respond to all items (*p* < 0.001). Of the adolescents responding to all items of the ecSI 2.0, 37% were in primary and 67% in secondary school. Of those who did not respond to all items, 63% were in primary school and 33% in secondary school.

This study was approved by the Ethics Committee of University of Eastern Finland. School principals informed adolescents and their parents about the study. The adolescents and their parents were given the possibility to refuse participation, which no one did.

### 2.2. Study Questionnaire

The study questionnaire included items (all in Finnish) on eating competence, eating patterns, perception of body image, weight loss efforts, self-esteem and SOC (the study questionnaire is available on request from the authors). The participants also reported their body height and weight on the questionnaire. BMI was calculated (kg m^−2^). Classification into normal-weight, overweight or underweight was done according to the international BMI cut-offs for children [30,31].

*Eating competence* was measured by using the preliminary Finnish translation of ecSatter Eating Competence Inventory 2.0 (ecSI 2.0) [4,5]. The inventory is comprised of 16 items representing the four components of ecSatter (four items for each component). The response scale of ecSI 2.0 is comprised of four-points, *i.e.*, often, fairly often, sometimes, rarely or never, scored as 3, 2, 1, 0 and 0, respectively. The theoretical range of the sum score was from 0 to 48. In earlier studies in adults, an ecSI 2.0 score of 32 or higher has been defined as the cut-off value for eating competence [3,4,5]. Despite the lack of data on eating competence in this age group, this same cut-off value was used in the present study in order to enable the comparability of the results with the earlier studies on eating competence. Thus those scoring high (scores ≥ 32) were classified to be “presumably eating competent” (pEC) and those who did not score high (scores < 32) as “presumably not eating competent” (not pEC). Due to the preliminary nature of the study, the terms “eating competent” and “not eating competent” were not used.

In the study questionnaire, 42 items concerned food selection and meal patterns. The items concerning type and frequency of meals and consumption of various types of foods have been used earlier in the questionnaires of the World Health Organization (WHO) [32] and the National Institute for Health and Welfare in Finland [33], both of which have been widely used among 11–17 years old children and adolescents in Finland [15,16]. Meals consumed during schooldays and weekends were evaluated on a four-point scale: never, 1–2 times per week, 3–4 times per week, and daily. Frequencies of meals consumed on school days and on weekends were combined to form a meal frequency sum variable (Cronbach’s alpha 0.79). Family meal frequency was evaluated on a five-point scale: Never, 1–2 times per week, 3–4 times per week, 5–6 times per week, and daily. Food consumption frequencies were evaluated in eight categories: (1) fast foods (consisting of pizza, hamburgers, hot dogs, kebabs, fried potatoes, French fries, meat pies and pastries, sum variable, Cronbach’s alpha 0.90); (2) porridge; (3) rye and crisp bread; (4) milk and sour milk; (5) salty and sweet snacks (consisting of popcorn, salted nuts, chips, chocolate, sweets, buns, cookies and doughnuts, sum variable, Cronbach’s alpha 0.78); (6) vegetables, (7) fruits; and (8) energy-containing beverages (consisting of soft drinks, energy drinks, and hot chocolate, sum variable, Cronbach’s alpha 0.65). Responses were indicated on a seven-point scale; never, rarely (1–2 times a month), once a week, every second day (2–4 times a week), almost daily (5–6 times a week), daily, and several times a day. The frequency of snacks was measured on a five-point scale: four or more snacks a day, three snacks a day, one to two snack a day, does not eat snacks daily and hardly ever eats snacks. The responses for snacking frequency were then formed to comprise three groups: (1) “four or more snacks a day” and “three snacks a day”; (2) “one to two snacks a day”; (3) “does not eat snacks daily” and “hardly ever eats snacks”. The option of one to two snacks a day was regarded as an optimal one according to the recommendation of The Finnish National Nutrition recommendations [34]. Family food selection and meal patterns were measured by responses to 16 statements on the availability of vegetables, fruit, salty snacks, sweet snacks and soft drinks, the child’s possibility to influence food choices and eating at home, and whether the child took part in food preparation. Responses were given on a five-point Likert scale ranging from “fully disagree” (1) to “fully agree” (5). In order to dichotomize the responses for statistical analyses, the responses “agree” and “somewhat agree” were combined as “agree” and the responses “cannot say”, “somewhat disagree” and “disagree” as “disagree”.

The *perception of body size* was evaluated with the options: somewhat fat, too fat, appropriate size, somewhat thin and too thin [32]. The questionnaire also included an item about possible weight-loss efforts during the previous 12 months [32].

*Self-esteem* was evaluated using the Rosenberg’s Self-Esteem Scale (RSE) [35], which has been considered to be a reliable and valid tool for the assessment of self-esteem among adolescents [36]. The scale comprises ten statements on self-satisfaction, self-worth and self-respect. Responses were given on a four-point Likert scale ranging from “fully disagree” (1) to “fully agree” (4). The theoretical range of the RSE sum score is from 10 to 40, with higher scores indicating higher self-esteem. Tertiles according to the sum scores were calculated. The cut-offs for the RSE tertiles were 10 to 25 for low, 26 to 30 for moderate, and 31 to 40 for high. Cronbach’s alpha for RSE was 0.72.

*Sense of coherence (SOC)* was monitored using the 13-item scale of Antonovsky [23], which has been shown to be applicable to children aged 12 years and older [37]. In our previous study using the SOC-13 scale, the results of children aged 10–11 were comparable to those obtained among children of ages 12 and older [29]. The theoretical range of the sum scores is from 13 to 91, with higher scores indicating stronger SOC. Tertiles according to the sum scores were calculated. The cut-offs for the SOC-13 tertiles were 17 to 52 for weak, 53 to 60 for moderate, and 62 to 91 for strong. Cronbach’s alpha for SOC-13 was 0.80.

### 2.3. Statistical Analyses

Statistical analyses were conducted with AMOS (SPSS Amos, 21.0) and SPSS (SPSS for WINDOWS, SPSS, 19.0, Chicago, IL, USA) software. Confirmatory factor analysis (CFA) was conducted on items measuring eating competence to examine the construct validity of ecSI 2.0. To determine the goodness-of-fit of the model, chi-square (χ2), the comparative fit index (CFI), the Tucker-Lewis index (TLI), the root mean square error of approximation (RMSEA), and the standardized root mean square residual (SRMR) indices were used. A non-significant χ2 indicates that the model has an acceptable fit for the data. Values greater than 0.90 for CFI and TLI, less than 0.08 for RMSEA, and SRMR indicate an acceptable model fit [38]. Internal consistencies of the sum variables were analyzed using Cronbach’s alpha coefficients of at least 0.60 as a criterion. Cross-tabulated frequencies between eating competence and the variables describing eating patterns, perception of body image, weight loss efforts, and the self-esteem and SOC tertiles were analyzed with the χ2-test. Mann Whitney U-test was used to compare ecSI 2.0 scores between boys and girls. Internal associations between presumed eating competence, self-esteem and SOC were analyzed by Spearman correlations. The Poisson regression model was used to assess the potential factors underlying eating competence. First, each explanatory variable was tested separately (univariate models), and then a multivariate model was conducted for the significant variables. The significant factors in the final model are reported as prevalence ratios (PRs) and their 95% confidence intervals (CIs). In the statistical analyses, a *p*-value < 0.05 was regarded as statistically significant.

## 3. Results

### 3.1. Construct Validity of ecSI 2.0

**Figure 2:**
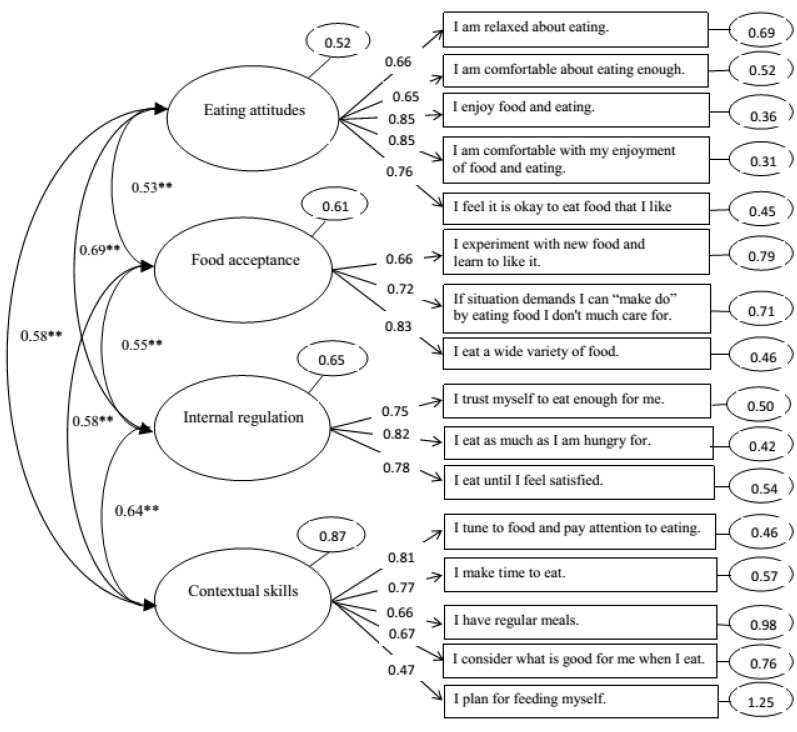
Measurement model (confirmatory factor analysis (CFA)) of the preliminary Finnish translation of ecSatter Eating Competence Inventory 2.0 (ecSI 2.0) (*n* = 976). Two-headed arrows between ellipses represent correlations between ecSI 2.0 main components. ** Correlations are significant at the 0.001 level. Arrows to the right indicate standardized regression weights between main components and individual items of the ecSI 2.0. Residual variances (unstandardized) for each main component and item are presented in ellipses after these.

In the CFA, the model fit for the four-factor model was acceptable (χ^2^ (98) = 769.155, *p* < 0.001, CFI = 0.91, TLI = 0.93, RMSEA = 0.080, SRMR = 0.066) (Figure 2). All four components of the ecSatter in the ecSI 2.0 correlated with each other (all *p*-values < 0.001). Cronbach’s alpha for the ecSI 2.0 was 0.92 and for its subscales the alphas were: eating attitudes 0.87, food acceptance 0.78, internal regulation 0.83 and contextual skills 0.81.

### 3.2. Eating Competence and Related Psychobehavioral Factors

The ecSI 2.0 scores ranged from 0 to 48 (mean 31.5, SD 11.6; median 34). The percentage distributions of responses to each item are reported in Table 1. Altogether 58% (*n* = 562) of the adolescents scored high (≥32) and were classified as “presumably eating competent” (pEC) (Table 2). Girls were more often pEC than boys (*p* = 0.006) and girls scored higher than boys (33 ± 10.9 *vs.* 30 ± 12.2, mean ± SD, *p* = 0.003). Proportions of pEC and not pEC adolescents did not differ between different school grades. The pEC adolescents more frequently perceived their body size as appropriate (*p* < 0.001) and had less often tried to lose weight during the previous year (*p* < 0.001). Self-reported weight status did not differ between the groups. Eating competence was significantly associated with self-esteem (*r* = 0.32, *p* < 0.001) and SOC (*r* = 0.27, *p* < 0.001). Self-esteem and SOC were also considerably intercorrelated (*r* = 0.47, *p* < 0.001).

**Table 1:** Percentage (%) distribution of the adolescents’ responses to the preliminary Finnish translation of the ecSatter Eating Competence Inventory 2.0 (*n* = 976).

	Never/Rarely	Some-Times	Often/Fairly Often
	←%→		
**Eating Attitudes**			
I am relaxed about eating.	8	26	66
I am comfortable about eating enough.	4	15	81
I enjoy food and eating.	10	22	68
I am comfortable with my enjoyment of food and eating.	7	20	73
I feel it is okay to eat food that I like.	5	18	77
**Food Acceptance**			
I experiment with new food and learn to like it.	16	29	55
If the situation demands, I “can make do” by eating food I don`t much care for.	16	33	51
I eat a wide variety of food.	15	23	62
**Internal Regulation**			
I trust myself to eat enough for me.	6	24	70
I eat as much as I am hungry for.	10	20	70
I eat until I feel satisfied.	12	24	64
**Contextual Skills**			
I tune in to food and pay attention to eating.	12	24	64
I make time to eat.	15	28	57
I have regular meals.	19	28	53
I consider what is good for me when I eat.	13	28	59
I plan for feeding myself.	38	29	33

### 3.3. Associations between Eating Competence and Eating Patterns

**Figure 3:**
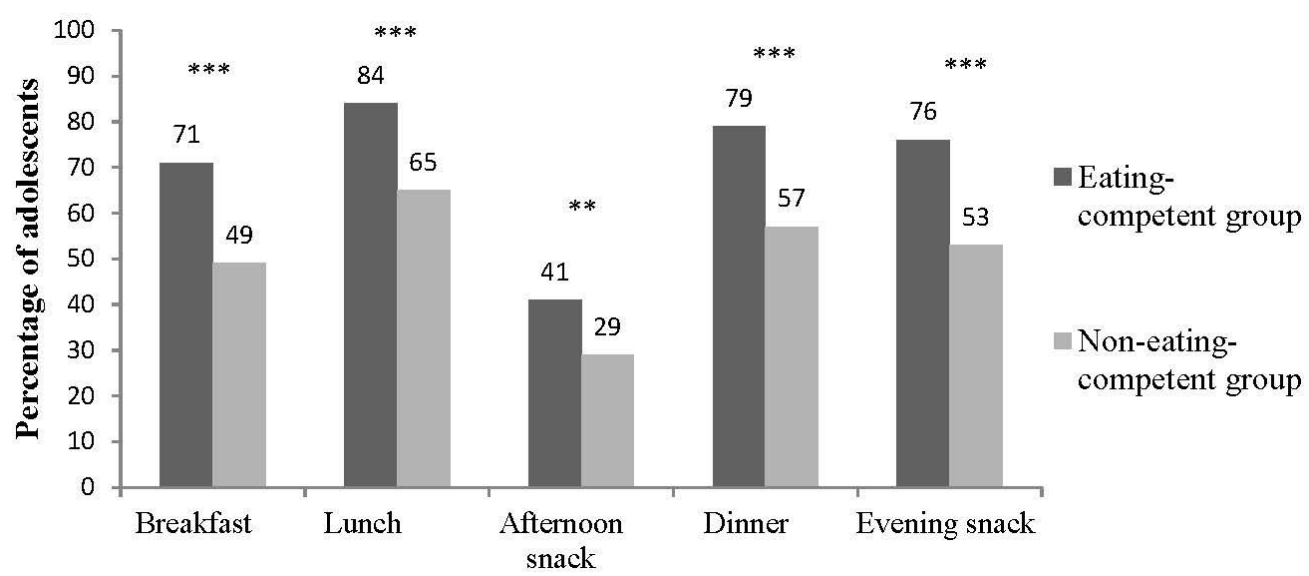
Meal frequency during school days in the presumably eating competent (pEC) and not eating competent (not pEC) groups (percentage of adolescents who consumed meals daily, *n* = 961–974 out of 976). Frequencies were generated by cross-tabulations using chi-square test for statistical significance (** *p* < 0.01, *** *p* < 0.001).

Presumed eating competence was associated with higher meal frequency both on school days (all *p*-values < 0.01, Figure 3) and at weekends (data not shown, all *p*-values < 0.001), and with lower overall snacking frequency (data not shown, *p* < 0.001). Frequent daily snacking (three or more snacks daily) was more common in the not pEC (32% of pEC *vs.* 47% of not pEC) whereas the pEC adolescents more often consumed one or two snacks daily (52% of pEC *vs.* 34% of not pEC) (*p* < 0.001).

Presumed eating competence was associated with the overall quality of diet. The pEC adolescents more often consumed vegetables (*p* < 0.001), fruits (*p* = 0.039), milk or sour milk (*p* < 0.001), porridge (*p* = 0.003) and rye and crisp bread (*p* < 0.001) and less often fast food (*p* < 0.001), salty and sweet snacks (*p* < 0.001) and energy-containing beverages (*p* < 0.001) than the not pEC adolescents.

**Figure 4:**
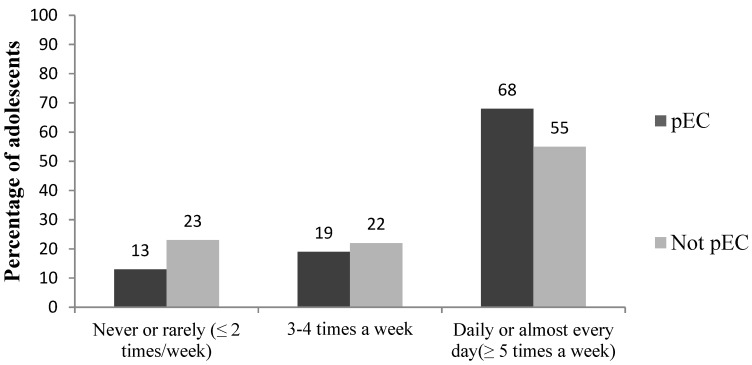
Family meal frequency in the presumably eating competent (pEC) and not eating competent (not pEC) groups (percentage of adolescents, *n* = 971 out of 976). Frequencies were generated by cross-tabulations using chi-square test for statistical significance, *p* < 0.001.

The pEC adolescents more often had regular family meal frequencies, more frequently had vegetables and fruit available at home, and their parents paid more attention to the quality of diet, as compared to the not pEC ones (all *p*-values < 0.05, Table 3, Figure 4). The pEC adolescents also reported more often that they could influence the selection and preparation of food as well as their own eating at home compared to the not pEC ones.

**Table 2:** Characteristics of the adolescents who scored high (scores ≥ 32 in the preliminary Finnish translation of the ecSatter Eating Competence Inventory 2.0) and were classified as “presumably eating competent” (pEC) (*n* = 582, 58% of adolescents).

Characteristic	pEC	*p*-Value ^1^
	% (CI), Number of Subjects	
Girls	62 (58–66), *n* = 324	
Boys	53 (48–58), *n* = 230	
		0.006
Primary school students	63 (57–68), *n* = 202	
Secondary school students	57 (53–62), *n* = 319	
		0.14
Overweight ^2^	60 (51–69), *n* = 67	
Normal weight ^2^	63 (58–67), *n* = 327	
Underweight ^2^	52 (40–65), *n* = 33	
		0.45
Perception of body image		
appropriate size	63 (59–69), *n* = 350	
somewhat fat or too fat	47 (41–53), *n* = 126	
somewhat thin or too thin	59 (50–68), *n* = 73	
		< 0.001
Tried to lose weight during last year	47 (41–52), *n* = 140	
		< 0.001
High self-esteem ^3^	77 (72–82), *n* = 234	
Moderate self-esteem ^3^	51 (45–56), *n* = 158	
Low self-esteem ^3^	44 (38–50), *n* = 115	
		< 0.001
Strong SOC (scores 62–91) ^4^	76 (69–81), *n* = 146	
Moderate SOC (scores 53–60) ^4^ 42 (35–49), *n* = 83		
Weak SOC (scores 17–52) ^4^	45 (39–52), *n* = 94	
		< 0.001

^1^ Frequencies were generated by cross-tabulations using chi-square test for statistical significance. ^2^ Mean body mass index (BMI) in normal weight group was 19 kg m^−2^, in overweight group 27 kg m^−2^ and in underweight group 16 kg m^−2^. ^3^ Self-esteem was measured by using Rosenberg’s Self-Esteem scale and divided into tertiles according to linear scores based on this scale. ^4^ Sense of coherence (SOC) was measured by using the Sense of Coherence 13 Scale and divided into tertiles according to linear scores based on this scale.

**Table 3:** Family eating patterns and adolescents’ opportunity to influence food and eating at home in the presumably eating competent (pEC) and not eating competent (not pEC) groups (*n* = 963–973 out of 976). Percentage (%) of adolescents who “agree” or “somewhat agree” with the item.

	pEC	not pEC	*p*-Value ^1^
Statement	←%→		
Family eating patterns			
In our family we have regular meal frequency.	82	60	< 0.001
We have vegetables included in every family meal.	83	54	< 0.001
In our family fruits are offered daily.	91	68	< 0.001
My parents pay attention to the quality of the diet.	93	73	< 0.001
We don’t usually have salty snacks available at home.	67	62	0.64
We don’t usually have sweet snacks available at home.	68	60	0.08
We don’t usually have soft drinks available at home.	67	71	0.005
Children’s possibility to influence food and eating			
I can influence the type of food eaten at home.	93	77	< 0.001
I can influence when I eat at home.	74	69	0.11
I can influence what I eat at home.	82	61	< 0.001
I can influence how much I eat at home.	94	82	< 0.001
I take part in food preparation at home.	73	46	< 0.001

^1^ Frequencies were generated by cross-tabulations using chi-square test for statistical significance.

### 3.4. Factors Associated with Eating Competence

According to the requirements of the Poisson regression model, only those who had responded to all the items in the study questionnaire were included in the statistical analysis (*n* = 538). The adolescents responding to all items did not differ in gender, school grade, weight status, perception of body size or self-esteem or SOC from those who had not responded all items (*n* = 436).

According to the univariate Poisson regression models, meal frequency, family eating patterns (availability of vegetables and fruit at home, parents’ attention to the healthfulness of food, the items describing the adolescent’s possibility to influence food and eating at home), gender, self-esteem and SOC were all significantly related to presumed eating competence (all *p*-values < 0.05). The pEC adolescents were more likely to have higher frequency of meals, more health-promoting family eating patterns and greater possibility to influence food and eating at home. Also female gender, higher self-esteem and stronger SOC enhanced the likelihood of being pEC.

According to the final multivariate model conducted for all significant variables, meal frequency and family eating patterns (adolescent’s possibility to influence how much to eat at meals, availability of vegetables at family meals) (all *p*-values < 0.05) and self-esteem as a trend (*p* = 0.059) were significant factors related to presumed eating competence (Table 4).

## 4. Discussion

### 4.1. Usefulness of Eating Competence Concept

To the best of our knowledge, this is the first study in which the utility of the eating competence concept and applicability of the ecSI 2.0 instrument has been investigated in adolescents. The construct validity of the preliminary Finnish translated version of the ecSI 2.0 was affirmed. CFA results indicated an acceptable model fit, and all four components of the ecSI 2.0 strongly correlated with each other and had a high internal consistency. The results were also well in line with the earlier findings in adult [3,8,10,11] and college student populations [39]. When using the validated cut-off value of ≥32 for adults [3,4], 58% of the adolescents in the present study were classified as presumably eating competent. This proportion is well in line with previous results according to which 45%–59% of adult populations can be considered as competent eaters [8,9,11]. The associations found here between adolescents’ presumed eating competence and meal patterns, such as meal frequency and perceived body size and self-esteem, have also been reported earlier in adults [3,11]. The results on food selection and meal patterns were also well in line with those found in previous studies using the same instruments [15,16]. Furthermore, the results indicating that only one third of the adolescents reported that they planned for feeding themselves and only half that they were open to having new foods in their diet, can be considered to be quite typical for adolescents in this age group [40]. Similarly, the fact that there was no difference between pEC and not pEC adolescents in perception of ability to influence when food is eaten at home could also be regarded typical for this age group. Thus, coming back to the original study aim, it can be concluded that the eating competence concept can be useful in 10–17 year old Finnish adolescents. These findings are, however, preliminary and need to be confirmed in future studies by using an instrument validated for this age group.

**Table 4:** Factors related to presumed eating competence ^1^, multivariate Poisson regression model (*n* = 540 out of 976 ^2^).

Variable	PR	95% CI	*P*-Value
Possibility to influence how much to eat at meals ^3^	1.237	1.070–1.428	0.004
Meal frequency ^4^	1.017	1.004–1.030	0.009
Availability of vegetables at family meals ^3^	1.165	1.037–1.310	0.010
Self-esteem ^5^	1.010	1.000–1.021	0.059

^1^ defined as scoring ≥ 32 in the preliminary Finnish translation of the ecSatter Eating Competence Inventory 2.0. ^2^ All adolescents who responded to all items were included in the model. ^3^ Adolescents who “agree” were compared to those who “disagree” with the claim. ^4^ Continuous variable. Change compared per 1 unit. The sum score ranges from 10 to 35, with high scores indicating high meal frequency. ^5^ Continuous variable. Change compared per 1 unit. The sum score ranges from 10 to 40, with high scores indicating high self-esteem.

### 4.2. Eating Competence and Family Eating Patterns

The importance of family in the development of children’s eating patterns has been demonstrated previously [19,41,42]. Interestingly, in the present study the availability of vegetables at family meals was significantly related to presumed eating competence. It cannot be assumed that merely increasing the availability of vegetables at family meals enhances eating competence. Greater availability of vegetables is probably indicative of more general features of family’s eating patterns and attitudes. In line with this assumption, almost all pEC adolescents (93%) reported that their parents paid attention to the quality of the diet. The adolescents’ possibility to influence how much to eat at family meals was another factor strongly associated with being pEC. This is in line with the Satter feeding dynamics model (fdSatter), which states that children are responsible for how much to eat, the parents for what, when and where to eat [43]. Furthermore, in previous studies parental feeding style exerting a high level of control over children’s eating has been associated with reduced likelihood to consume fruit and vegetables [44]. The controlling feeding style is also harmful to appetite control by encouraging children to eat according to external rather than internal cues [45,46]. Thus, a parental feeding style which supports children’s own capability by encouraging them to regulate their eating based on appetite responses, as well as by offering them opportunities to take part in food choice and cooking, may strengthen children’s eating competence. It should also be considered that other factors, such as demographic factors (income, occupation, education level etc.) influence family eating patterns, e.g., whether families eat together, and they could thus act as mediating factors when thinking about the possible associations between eating competence and eating patterns.

### 4.3. Eating Competence, Weight and Body Satisfaction

The ecSatter model emphasizes satisfaction with body weight regardless of real body weight [1]. In line with this, the pEC adolescents more often perceived their body size as appropriate. On the contrary, the not pEC adolescents more often perceived their body as somewhat or too fat, despite the fact that the self-reported weight status did not differ between these two groups. In addition, almost half of those in the not pEC group had tried to lose weight during the past year. Body dissatisfaction is a risk factor for overweight and eating disorders [47,48,49]. Thus these results also have clear clinical importance. Moreover, regular meal frequency, as an essential component of contextual skills, and healthier eating patterns in general, have been associated with decreased risk for overweight [50]. Regular meal frequency, especially consumption of breakfast, has been shown to be protective even in individuals with inherited vulnerability for obesity [51]. Presumed eating competence was also associated with greater consumption of vegetables and fruit, which in turn has been found to protect against overweight as well as to be associated with better health in the long run [52].

### 4.4. Eating Competence, Self-Esteem and SOC

Presumed eating competence was associated with high self-esteem and strong SOC. As various items in the ecSI 2.0 refer to self-confidence (e.g., “I am comfortable with my enjoyment of food and eating”, “I trust myself to eat enough for me”), the association is logical and also supported by earlier findings in adult populations [3,4]. Moreover, having high self-esteem and experiencing life as more comprehensive, manageable and meaningful, as expressed by the subcomponents of SOC, may reflect a higher level of caring about one’s health and well-being, which in turn could correlate with eating competence. This could mean that in order to support adolescents’ eating competence, attention should be paid to self-esteem and SOC though the direction of the relation could also be the opposite way.

### 4.5. Eating Competence and Gender

The finding that girls were more often presumably competent eaters than boys was opposite to that reported earlier in college students [39]. This is a novel finding and needs to be confirmed in further studies. One possibility is that it is related to the earlier maturation of girls than boys [53].

### 4.6. Strengths and Limitations

The strength of the present study is the large number of study subjects. Furthermore, it could be argued that there was hardly any selection bias due to socioeconomic or other demographic factors as the study was performed in schools within the general Finnish education system. The relatively high proportion of missing data due to incompletely filled in items in the study questionnaire could have potentially biased the responses. There were, however, no significant differences in gender, weight status, perception of body size, self-esteem and SOC between those who filled in all the items in the ecSI 2.0 and those who did not. These two groups differed only with respect to age. Adolescents who responded to all the items in the ecSI 2.0 were older than those who did not respond to all the items.

As this is the first study using the ecSI 2.0 in this age group, the results should be interpreted with caution. The current study utilized the preliminary Finnish translation of the same instrument that has been used earlier in adults. The cut-off value used for presumed eating competence was also the same as has been set before for adults. Therefore, it remains to be determined in further studies whether this cut-off score is suitable also for adolescents. The main limitation of the study has to do with the face validation of ecSI 2.0; it was not confirmed by cognitive interviewing techniques. Therefore, it is unsure whether the phenomenon measured by the ecSI 2.0 in adolescents corresponds to eating competence as the same construct as in adults. Moreover, in the ecSI 2.0 in particular the questions on contextual skills are potentially problematic, since taking care of meals is mainly a parental responsibility. It is possible that adolescents may not have described their own agency. This might have been the case especially with the statement “I plan for feeding myself”, which also showed the lowest proportion of “often/fairly often” responses of all the statements in the ecSI 2.0. Nevertheless, the study revealed, novel and consistent, although preliminary findings on the factors playing a role in food selection and meal patterns in adolescents.

The lack of validation of the original content of ecSI 2.0 by back translation prior to the study can be regarded as the second shortcoming of the study. However, back translation, carried out retroactively, did not bring out any relevant differences between the back translated version and the version applied in the study. However, in future studies, an instrument with final approved back translation is needed to confirm the validity of the findings. Thirdly, slightly modified response options as compared with the original survey (option “often” instead of “always”, option “fairly often” instead of “often”) were a shortcoming of the study. However, all the main results of the present study were consistent with those in our more recent study [54]. In brief, 10–17 year old Finnish adolescents (*n* = 427) filled in the same study questionnaire with the original response options. As a result, the ecSI 2.0 scores ranged from 0 to 48 (mean 29.4). The pEC was significantly associated with greater meal frequency and more frequent consumption of vegetables, fruits and berries. In addition, pEC was associated with the perception of body size as appropriate and with less weight loss efforts. The pEC adolescents also had more frequent and health-promoting family meals and meal patterns. It is thus likely that the potential effect of the use of slightly different response options in the current study remains negligible. Fourthly, cross-sectional study design is not ideal for the investigation of possible determinants of eating competence. Height and weight data were based on self-reports. This could have contributed to the bias of underreporting of body weight and overestimation of body height. If so, this would have caused the underestimation of the prevalence of overweight and obesity [55,56]. On the other hand, self-reported and measured weights have been shown to correlate highly in adolescents [56,57]. Self-reported data has also been concluded to be valuable as the only source of data [58]. The age range of the study population was also large. Therefore, in further studies eating competence needs to be studied by using a prospective study design to better understand its development along the maturation of children and adolescents. The results of the present study also need to be confirmed by other similar study populations.

## 5. Conclusions

Eating competence, as defined and measured by the preliminary Finnish translated version of ecSI 2.0, seems to be a useful concept in adolescents and is associated with health-promoting food selection and meal patterns, perception of body size as appropriate, higher self-esteem and stronger SOC. Encouraging frequent family meals and autonomy in one’s own control over eating, as well as the other tenets of the ecSatter model, could be important targets for health promotion of adolescents.
